# 2-[4-(4,5-Dihydro-1*H*-imidazol-2-yl)phen­yl]-4,5-dihydro-1*H*-imidazol-3-ium 4-amino­benzoate

**DOI:** 10.1107/S1600536810051202

**Published:** 2010-12-18

**Authors:** Xiu-Mei Song, Jun-Jun Li, Xin-Hua Liu, Chun-Xia Ren, Shao-Ming Shang

**Affiliations:** a School of Chemical and Material Engineering, Jiangnan University, 1800 Lihu Road, Wuxi, Jiangsu Province 214122, People’s Republic of China; b College of Pharmacy, GuangDong Pharmaceutical University, Guangzhou, Guangdong Province 510006, People’s Republic of China

## Abstract

In the cation of the title compound, C_12_H_15_N_4_^+^·C_7_H_6_NO_2_^−^, the benzene ring makes dihedral angles of 30.51 (9) and 25.64 (9)° with the imidazole and imidazolinium rings, respectively. In the crystal, inter­molecular N—H⋯O and N—H⋯N hydrogen-bonding inter­actions link the mol­ecules into a three-dimensional network.

## Related literature

For general background to supra­molecular inter­actions, see: Jeffrey (1997 ▶). For the structures of related metal complexes with imidazole ligands reported by our group, see: Ren, Ye, He *et al.* (2004 ▶); Ren, Ye, Zhu *et al.* (2004 ▶); Ren *et al.* (2007 ▶, 2009 ▶).
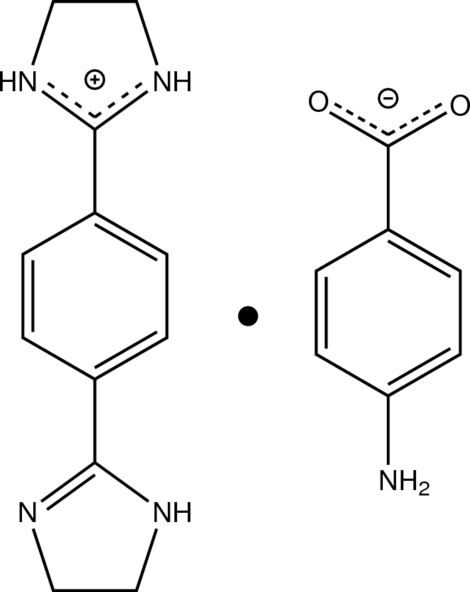


## Experimental

### 

#### Crystal data

C_12_H_15_N_4_^+^·C_7_H_6_NO_2_^−^
*M* *_r_* = 351.41
Monoclinic, 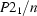

*a* = 7.5006 (15) Å
*b* = 29.031 (6) Å
*c* = 7.9361 (16) Å
β = 95.54 (3)°
*V* = 1720.0 (6) Å^3^
*Z* = 4
Mo *K*α radiation
μ = 0.09 mm^−1^
*T* = 293 K
0.75 × 0.62 × 0.51 mm

#### Data collection

Bruker SMART APEX CCD diffractometer
Absorption correction: multi-scan (*SADABS*; Bruker, 1998 ▶) *T*_min_ = 0.934, *T*_max_ = 0.955
9730 measured reflections
3381 independent reflections
1911 reflections with *I* > 2σ(*I*)
*R*_int_ = 0.061

#### Refinement

*R*[*F* ^2^ > 2σ(*F* ^2^)] = 0.055
*wR*(*F* ^2^) = 0.146
*S* = 0.98
3381 reflections
236 parameters
H-atom parameters constrained
Δρ_max_ = 0.24 e Å^−3^
Δρ_min_ = −0.24 e Å^−3^

### 

Data collection: *SMART* (Bruker, 1998 ▶); cell refinement: *SAINT-Plus* (Bruker, 1998 ▶); data reduction: *SAINT-Plus*; program(s) used to solve structure: *SHELXS97* (Sheldrick, 2008 ▶); program(s) used to refine structure: *SHELXL97* (Sheldrick, 2008 ▶); molecular graphics: *SHELXTL* (Sheldrick, 2008 ▶); software used to prepare material for publication: *SHELXTL*.

## Supplementary Material

Crystal structure: contains datablocks global, I. DOI: 10.1107/S1600536810051202/rz2530sup1.cif

Structure factors: contains datablocks I. DOI: 10.1107/S1600536810051202/rz2530Isup2.hkl

Additional supplementary materials:  crystallographic information; 3D view; checkCIF report

## Figures and Tables

**Table 1:** Hydrogen-bond geometry (Å, °)

*D*—H⋯*A*	*D*—H	H⋯*A*	*D*⋯*A*	*D*—H⋯*A*
N5—H5*A*⋯O2^i^	0.86	1.86	2.719 (3)	174
N4—H4*A*⋯N2^ii^	0.86	2.25	3.059 (3)	156
N3—H3*A*⋯N1^iii^	0.86	2.20	3.035 (3)	165
N1—H1*B*⋯O1^iv^	0.86	2.15	2.972 (3)	160
N1—H1*A*⋯O2^v^	0.86	2.12	2.962 (3)	166

Symmetry codes: (i) 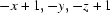; (ii) 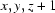; (iii) 
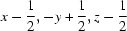
; (iv) 
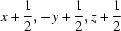
; (v) 
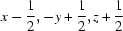
.

**Fig. 1.**
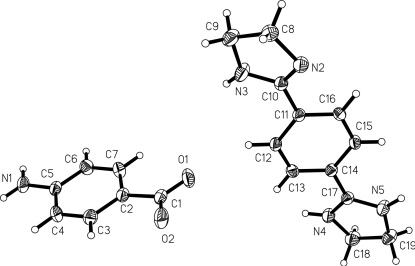
The molecular structure of the title compound showing 30% probability displacement ellipsoids.

**Fig. 2.**
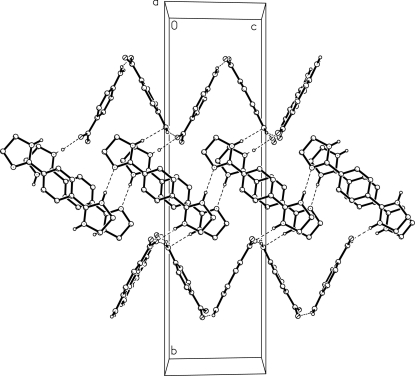
Crystal packing of the title compound viewed along the a axis. H atoms not involved in hydrogen bonding are omitted for clarity.

## supplementary crystallographic information

### Comment

Attention has been recently focused on the use of supramolecular interactions, such as hydrogen bonding and π-π stacking interactions, in the controlled assembly of supramolecular architectures (Jeffrey, 1997). Hydrogen bonds often play a dominant role in crystal engineering because of they combine strength with directionality. On the other hand, supramolecular systems sustained by such soft connections are comparatively more flexible and sensitive to the chemical environment. Consequently, hydrogen bond sustained systems are less designable and remain to be further investigated. We have reported several complexes having an imidazole entity, and have concluded that hydrogen bonding involving this group influences the geometry around the metal atom and the crystallization mechanism (Ren, Ye, He *et al.*, 2004; Ren, Ye, Zhu *et al.*, 2004; Ren, *et al.*, 2007; Ren, *et al.*, 2009). As a further contribution to this field, we describe herein the synthesis and crystal structure of the title compound.

The asymmetric unit of the title compound (Fig. 1) contains one 1-(4,5-dihydro-1*H*,3*H*-imidazol-2-yl)-4-(4,5-dihydro- 1*H*-imidazolinium-2-yl)benzene cation ands one 4-aminobenzoate anion. In the cation, both the imidazole (N2/N3/C8—C10) and imidazolinium rings adopt an envelope conformation, with atoms C11 and C14 displaced by -0.048 (2) and 0.018 (2) Å, respectively, from plane of the other ring atoms. The dihedral angle they form with the benzene ring is 30.51 (9) and 25.64 (9)°, respectively. In the crystal structure, intramolecular N—H···O and N—H···N hydrogen interactions (Table 1) link the molecules into a three-dimensional network (Fig. 2).

### Experimental

All the reagents and solvents employed were commercially available and used as received without further purification. Synthesis of 1,4-bis(4,5-dihydro-1*H*-imidazol-2-yl)benzene: a mixture of 1,4-benzenedicarboxylic acid (2.31 g, 13.9 mmol), ethylenediamine (3.70 ml, 50 mmol), ethylenediamine dihydrochloride (6.64 g, 50 mmol) and toluene-*p*-sulfonic acid (0.208 g, 1.09 mmol) in ethyleneglycol (20 ml) was refluxed at 198°C for 3 h. About half of the ethylene glycol solvent was then slowly removed by distillation at 120°C. The residue was dissolved in a mixture of water (40 ml) and concentrated hydrochloric acid (11 M, 3 ml). The addition of 50% aqueous sodium hydroxide gave a yellow precipitate that was recrystallized by methanol (yield 83% based on 1,4-benzenedicarboxylic acid; *ca* 2.50 g). Calc. for C_12_H_14_N_4_: C 67.27; H 6.59; N 26.15%. Found: C 66.98; H 6.92; N 26.08%. IR (KBr, cm^-1^): 3188(m), 2936(m), 2866(m), 1606(s), 1532(s), 1466(s), 1345(m), 1270(s), 1191(w), 1080(w), 981(m), 855(m). Synthesis of the title compound: to a solution of 1,4-bis(4,5-dihydro-1*H*-imidazol-2-yl)benzene (0.0043 g, 0.02 mmol) in methanol (1 ml), an acetonitrile solution (1 ml) of 4-aminobenzoic acid (0.0027 g, 0.021 mmol) was added and stirred for 10 min at room temperature. Diethyl ether (10 ml) was then added and the solution was allowed to slowly evaporate at room temperature for 25 h. Colourless prismatic crystals of the title compound were obtained, which were collected by filtration, washed with water and dried in vacuum desiccator over silica gel (yield 0.0034 g, 39%). IR (KBr,cm^-1^): 3433(w), 3089(m), 2966(w), 1595(s), 1514(w), 1380(s), 1282(m), 675(m).

### Refinement

Anisotropic thermal parameters were applied to all nonhydrogen atoms. The organic hydrogen atoms attached to C atoms and N atom were fixed geometrically and treated as riding with C—H = 0.93 Å (aromatic) or 0.97 Å (methylene) and N—H = 0.86 Å with *U*_iso_(H) = 1.2 *U*_eq_(C or N).

### Crystal data

**Table:**

C_12_H_15_N_4_^+^·C_7_H_6_NO_2_^−^	*F*(000) = 744
*M**_r_* = 351.41	*D*_x_ = 1.357 Mg m^−^^3^
Monoclinic, *P*2_1_/*n*	Mo *K*α radiation, λ = 0.71073 Å
Hall symbol: -P 2yn	Cell parameters from 1044 reflections
*a* = 7.5006 (15) Å	θ = 2.7–20.3°
*b* = 29.031 (6) Å	µ = 0.09 mm^−^^1^
*c* = 7.9361 (16) Å	*T* = 293 K
β = 95.54 (3)°	Block, colourless
*V* = 1720.0 (6) Å^3^	0.75 × 0.62 × 0.51 mm
*Z* = 4	

### Data collection

**Table:**

Bruker SMART APEX CCD diffractometer	3381 independent reflections
Radiation source: fine-focus sealed tube	1911 reflections with *I* > 2σ(*I*)
graphite	*R*_int_ = 0.061
phi and ω scans	θ_max_ = 26.0°, θ_min_ = 1.4°
Absorption correction: multi-scan (*SADABS*; Bruker, 1998)	*h* = −9→8
*T*_min_ = 0.934, *T*_max_ = 0.955	*k* = −35→35
9730 measured reflections	*l* = −9→8

### Refinement

**Table:**

Refinement on *F*^2^	Secondary atom site location: difference Fourier map
Least-squares matrix: full	Hydrogen site location: inferred from neighbouring sites
*R*[*F*^2^ > 2σ(*F*^2^)] = 0.055	H-atom parameters constrained
*wR*(*F*^2^) = 0.146	*w* = 1/[σ^2^(*F*_o_^2^) + (0.0671*P*)^2^] where *P* = (*F*_o_^2^ + 2*F*_c_^2^)/3
*S* = 0.98	(Δ/σ)_max_ = 0.001
3381 reflections	Δρ_max_ = 0.24 e Å^−^^3^
236 parameters	Δρ_min_ = −0.24 e Å^−^^3^
0 restraints	Extinction correction: *SHELXL97* (Sheldrick, 2008), Fc^*^=kFc[1+0.001xFc^2^λ^3^/sin(2θ)]^-1/4^
Primary atom site location: structure-invariant direct methods	Extinction coefficient: 0.012 (2)

### Special details

**Table:**

Geometry. All e.s.d.'s (except the e.s.d. in the dihedral angle between two l.s. planes) are estimated using the full covariance matrix. The cell e.s.d.'s are taken into account individually in the estimation of e.s.d.'s in distances, angles and torsion angles; correlations between e.s.d.'s in cell parameters are only used when they are defined by crystal symmetry. An approximate (isotropic) treatment of cell e.s.d.'s is used for estimating e.s.d.'s involving l.s. planes.
Refinement. Refinement of *F*^2^ against ALL reflections. The weighted *R*-factor *wR* and goodness of fit *S* are based on *F*^2^, conventional *R*-factors *R* are based on *F*, with *F* set to zero for negative *F*^2^. The threshold expression of *F*^2^ > σ(*F*^2^) is used only for calculating *R*-factors(gt) *etc*. and is not relevant to the choice of reflections for refinement. *R*-factors based on *F*^2^ are statistically about twice as large as those based on *F*, and *R*- factors based on ALL data will be even larger.

### Fractional atomic coordinates and isotropic or equivalent isotropic displacement parameters (Å^2^)

**Table:**

	*x*	*y*	*z*	*U*_iso_*/*U*_eq_	
O1	0.5344 (3)	0.13642 (6)	0.6366 (3)	0.0639 (6)	
O2	0.8172 (3)	0.14561 (6)	0.6007 (3)	0.0609 (6)	
N1	0.6747 (3)	0.33128 (6)	0.9952 (3)	0.0499 (6)	
H1A	0.5753	0.3430	1.0205	0.060*	
H1B	0.7732	0.3462	1.0175	0.060*	
C1	0.6750 (4)	0.15795 (8)	0.6570 (3)	0.0390 (6)	
C2	0.6789 (3)	0.20287 (8)	0.7521 (3)	0.0384 (6)	
C3	0.8346 (3)	0.22767 (8)	0.7889 (3)	0.0454 (7)	
H3	0.9419	0.2160	0.7572	0.055*	
C4	0.8346 (3)	0.26964 (8)	0.8720 (3)	0.0465 (7)	
H4	0.9417	0.2855	0.8971	0.056*	
C5	0.6761 (3)	0.28824 (8)	0.9181 (3)	0.0395 (6)	
C6	0.5195 (3)	0.26380 (8)	0.8812 (3)	0.0478 (7)	
H6	0.4120	0.2756	0.9117	0.057*	
C7	0.5216 (3)	0.22201 (9)	0.7996 (3)	0.0475 (7)	
H7	0.4145	0.2061	0.7756	0.057*	
N2	0.2106 (3)	0.05786 (7)	−0.0118 (3)	0.0462 (6)	
N3	0.2120 (3)	0.11748 (7)	0.1670 (3)	0.0591 (7)	
H3A	0.2232	0.1309	0.2641	0.071*	
N4	0.2894 (3)	−0.03934 (7)	0.8663 (3)	0.0449 (6)	
H4A	0.3020	−0.0114	0.9013	0.054*	
N5	0.2594 (3)	−0.09627 (7)	0.6884 (3)	0.0479 (6)	
H5A	0.2384	−0.1102	0.5930	0.057*	
C8	0.1983 (4)	0.10061 (9)	−0.1168 (3)	0.0513 (7)	
H8A	0.0926	0.0997	−0.1975	0.062*	
H8B	0.3031	0.1037	−0.1786	0.062*	
C9	0.1869 (4)	0.14075 (9)	0.0057 (3)	0.0553 (8)	
H9A	0.2806	0.1632	−0.0060	0.066*	
H9B	0.0712	0.1559	−0.0103	0.066*	
C10	0.2153 (3)	0.07153 (8)	0.1422 (3)	0.0369 (6)	
C11	0.2280 (3)	0.04022 (7)	0.2894 (3)	0.0336 (6)	
C12	0.3128 (3)	0.05367 (8)	0.4445 (3)	0.0359 (6)	
H12	0.3630	0.0829	0.4569	0.043*	
C13	0.3230 (3)	0.02398 (8)	0.5803 (3)	0.0362 (6)	
H13	0.3801	0.0333	0.6839	0.043*	
C14	0.2486 (3)	−0.01969 (8)	0.5636 (3)	0.0333 (6)	
C15	0.1646 (3)	−0.03314 (8)	0.4082 (3)	0.0364 (6)	
H15	0.1165	−0.0626	0.3952	0.044*	
C16	0.1517 (3)	−0.00331 (7)	0.2732 (3)	0.0362 (6)	
H16	0.0917	−0.0123	0.1705	0.043*	
C17	0.2651 (3)	−0.05161 (8)	0.7064 (3)	0.0355 (6)	
C18	0.2921 (4)	−0.07978 (9)	0.9765 (3)	0.0536 (7)	
H18A	0.1868	−0.0808	1.0383	0.064*	
H18B	0.3985	−0.0802	1.0563	0.064*	
C19	0.2929 (4)	−0.11937 (9)	0.8510 (3)	0.0535 (8)	
H19A	0.4078	−0.1350	0.8606	0.064*	
H19B	0.1994	−0.1415	0.8678	0.064*	

### Atomic displacement parameters (Å^2^)

**Table:**

	*U*^11^	*U*^22^	*U*^33^	*U*^12^	*U*^13^	*U*^23^
O1	0.0623 (13)	0.0418 (11)	0.0858 (16)	−0.0113 (10)	−0.0017 (11)	−0.0208 (10)
O2	0.0679 (14)	0.0419 (11)	0.0755 (15)	−0.0065 (9)	0.0207 (11)	−0.0182 (10)
N1	0.0584 (15)	0.0315 (12)	0.0603 (16)	−0.0034 (10)	0.0084 (12)	−0.0091 (11)
C1	0.0540 (18)	0.0288 (14)	0.0343 (15)	0.0008 (12)	0.0044 (13)	0.0005 (11)
C2	0.0472 (15)	0.0319 (13)	0.0360 (15)	−0.0010 (11)	0.0033 (12)	−0.0005 (11)
C3	0.0486 (16)	0.0408 (15)	0.0473 (17)	0.0009 (12)	0.0070 (13)	−0.0034 (13)
C4	0.0500 (17)	0.0394 (15)	0.0496 (18)	−0.0103 (12)	0.0020 (13)	−0.0119 (13)
C5	0.0546 (16)	0.0280 (13)	0.0358 (15)	−0.0011 (12)	0.0029 (12)	−0.0013 (11)
C6	0.0478 (16)	0.0448 (16)	0.0511 (18)	0.0003 (12)	0.0069 (13)	−0.0105 (13)
C7	0.0487 (16)	0.0429 (15)	0.0505 (18)	−0.0097 (12)	0.0026 (13)	−0.0092 (13)
N2	0.0654 (15)	0.0399 (13)	0.0324 (13)	−0.0025 (10)	0.0002 (11)	0.0025 (10)
N3	0.109 (2)	0.0322 (12)	0.0354 (14)	0.0042 (12)	0.0051 (13)	0.0015 (10)
N4	0.0665 (15)	0.0360 (12)	0.0310 (13)	−0.0011 (10)	−0.0012 (11)	0.0019 (9)
N5	0.0720 (15)	0.0333 (12)	0.0386 (13)	0.0037 (11)	0.0063 (11)	−0.0002 (10)
C8	0.0670 (19)	0.0495 (17)	0.0364 (16)	−0.0061 (14)	0.0010 (13)	0.0092 (13)
C9	0.075 (2)	0.0439 (16)	0.0472 (19)	0.0056 (14)	0.0055 (15)	0.0099 (14)
C10	0.0416 (15)	0.0332 (14)	0.0355 (16)	0.0014 (11)	0.0018 (11)	−0.0011 (11)
C11	0.0380 (13)	0.0306 (13)	0.0319 (14)	0.0019 (11)	0.0024 (11)	−0.0023 (10)
C12	0.0427 (14)	0.0288 (13)	0.0361 (15)	0.0002 (10)	0.0023 (11)	−0.0039 (11)
C13	0.0399 (14)	0.0366 (14)	0.0312 (14)	0.0017 (11)	−0.0015 (11)	−0.0074 (11)
C14	0.0387 (14)	0.0312 (13)	0.0303 (14)	0.0026 (10)	0.0050 (11)	−0.0010 (11)
C15	0.0461 (15)	0.0303 (13)	0.0326 (15)	−0.0042 (11)	0.0024 (11)	−0.0040 (11)
C16	0.0435 (15)	0.0352 (14)	0.0284 (14)	−0.0001 (11)	−0.0037 (11)	−0.0043 (11)
C17	0.0391 (14)	0.0349 (14)	0.0327 (15)	0.0034 (11)	0.0045 (11)	−0.0018 (11)
C18	0.0703 (19)	0.0499 (17)	0.0400 (17)	0.0038 (14)	0.0018 (14)	0.0086 (13)
C19	0.0682 (19)	0.0416 (16)	0.0511 (19)	0.0075 (13)	0.0086 (15)	0.0112 (13)

### Geometric parameters (Å, °)

**Table:**

O1—C1	1.223 (3)	N5—C19	1.454 (3)
O2—C1	1.248 (3)	N5—H5A	0.8600
N1—C5	1.392 (3)	C8—C9	1.526 (3)
N1—H1A	0.8600	C8—H8A	0.9700
N1—H1B	0.8600	C8—H8B	0.9700
C1—C2	1.506 (3)	C9—H9A	0.9700
C2—C3	1.379 (3)	C9—H9B	0.9700
C2—C7	1.389 (3)	C10—C11	1.476 (3)
C3—C4	1.386 (3)	C11—C12	1.386 (3)
C3—H3	0.9300	C11—C16	1.388 (3)
C4—C5	1.387 (3)	C12—C13	1.376 (3)
C4—H4	0.9300	C12—H12	0.9300
C5—C6	1.379 (3)	C13—C14	1.386 (3)
C6—C7	1.376 (3)	C13—H13	0.9300
C6—H6	0.9300	C14—C15	1.386 (3)
C7—H7	0.9300	C14—C17	1.459 (3)
N2—C10	1.282 (3)	C15—C16	1.374 (3)
N2—C8	1.493 (3)	C15—H15	0.9300
N3—C10	1.349 (3)	C16—H16	0.9300
N3—C9	1.444 (3)	C18—C19	1.521 (4)
N3—H3A	0.8600	C18—H18A	0.9700
N4—C17	1.314 (3)	C18—H18B	0.9700
N4—C18	1.463 (3)	C19—H19A	0.9700
N4—H4A	0.8600	C19—H19B	0.9700
N5—C17	1.304 (3)		
			
C5—N1—H1A	120.0	N3—C9—H9A	111.5
C5—N1—H1B	120.0	C8—C9—H9A	111.5
H1A—N1—H1B	120.0	N3—C9—H9B	111.5
O1—C1—O2	124.2 (2)	C8—C9—H9B	111.5
O1—C1—C2	118.9 (2)	H9A—C9—H9B	109.3
O2—C1—C2	116.9 (2)	N2—C10—N3	116.5 (2)
C3—C2—C7	117.3 (2)	N2—C10—C11	123.9 (2)
C3—C2—C1	122.2 (2)	N3—C10—C11	119.6 (2)
C7—C2—C1	120.4 (2)	C12—C11—C16	119.2 (2)
C2—C3—C4	121.4 (2)	C12—C11—C10	121.2 (2)
C2—C3—H3	119.3	C16—C11—C10	119.5 (2)
C4—C3—H3	119.3	C13—C12—C11	120.3 (2)
C3—C4—C5	120.5 (2)	C13—C12—H12	119.8
C3—C4—H4	119.8	C11—C12—H12	119.8
C5—C4—H4	119.8	C12—C13—C14	120.5 (2)
C6—C5—C4	118.6 (2)	C12—C13—H13	119.8
C6—C5—N1	120.9 (2)	C14—C13—H13	119.8
C4—C5—N1	120.5 (2)	C15—C14—C13	119.1 (2)
C7—C6—C5	120.4 (2)	C15—C14—C17	120.6 (2)
C7—C6—H6	119.8	C13—C14—C17	120.2 (2)
C5—C6—H6	119.8	C16—C15—C14	120.5 (2)
C6—C7—C2	121.9 (2)	C16—C15—H15	119.7
C6—C7—H7	119.0	C14—C15—H15	119.7
C2—C7—H7	119.0	C15—C16—C11	120.3 (2)
C10—N2—C8	105.6 (2)	C15—C16—H16	119.9
C10—N3—C9	109.6 (2)	C11—C16—H16	119.9
C10—N3—H3A	125.2	N5—C17—N4	112.0 (2)
C9—N3—H3A	125.2	N5—C17—C14	123.2 (2)
C17—N4—C18	110.6 (2)	N4—C17—C14	124.8 (2)
C17—N4—H4A	124.7	N4—C18—C19	102.4 (2)
C18—N4—H4A	124.7	N4—C18—H18A	111.3
C17—N5—C19	111.1 (2)	C19—C18—H18A	111.3
C17—N5—H5A	124.4	N4—C18—H18B	111.3
C19—N5—H5A	124.4	C19—C18—H18B	111.3
N2—C8—C9	106.5 (2)	H18A—C18—H18B	109.2
N2—C8—H8A	110.4	N5—C19—C18	102.8 (2)
C9—C8—H8A	110.4	N5—C19—H19A	111.2
N2—C8—H8B	110.4	C18—C19—H19A	111.2
C9—C8—H8B	110.4	N5—C19—H19B	111.2
H8A—C8—H8B	108.6	C18—C19—H19B	111.2
N3—C9—C8	101.4 (2)	H19A—C19—H19B	109.1

### Hydrogen-bond geometry (Å, °)

**Table:**

*D*—H···*A*	*D*—H	H···*A*	*D*···*A*	*D*—H···*A*
N5—H5A···O2^i^	0.86	1.86	2.719 (3)	174.
N4—H4A···N2^ii^	0.86	2.25	3.059 (3)	156.
N3—H3A···N1^iii^	0.86	2.20	3.035 (3)	165.
N1—H1B···O1^iv^	0.86	2.15	2.972 (3)	160.
N1—H1A···O2^v^	0.86	2.12	2.962 (3)	166.

Symmetry codes: (i) −*x*+1, −*y*, −*z*+1; (ii) *x*, *y*, *z*+1; (iii) *x*−1/2, −*y*+1/2, *z*−1/2; (iv) *x*+1/2, −*y*+1/2, *z*+1/2; (v) *x*−1/2, −*y*+1/2, *z*+1/2.
